# Lactose-Free Dairy Products: Market Developments, Production, Nutrition and Health Benefits

**DOI:** 10.3390/nu11030551

**Published:** 2019-03-05

**Authors:** Peter J. T. Dekker, Damiet Koenders, Maaike J. Bruins

**Affiliations:** 1 DSM Biotechnology Center, Alexander Fleminglaan 1, 2613 AX Delft, The Netherlands; 2 DSM Food Specialties, Alexander Fleminglaan 1, 2613 AX Delft, The Netherlands; damiet.koenders@dsm.com; 3 Nutrition Science & Advocacy, DSM Nutritional Products, 4323 Kaiseraugst, Switzerland; maaike.bruins@dsm.com

**Keywords:** lactose-free, lactose intolerance, lactase, dairy products, nutrition economics

## Abstract

Lactose-free dairy is able to provide the essential nutrients present in regular dairy products, like calcium and vitamins, to those that are not able to digest lactose. This product category currently has a wide and growing health appeal to consumers. In recent years, the quality and product variety in the lactose-free dairy segment has been increasing significantly, giving consumers more tempting products to decide from. As a result, lactose-free dairy is now the fastest growing market in the dairy industry. This review discusses the market developments and production possibilities and issues related to the wide variation of lactose-free dairy products that are currently available. Additionally, the health benefits that lactose-free dairy may offer compared to dairy avoidance are illustrated.

## 1. Introduction

The percentage of people with an impaired ability to digest lactose varies widely per country and per continent, from 98–100% of adults in Southeast Asia to just 1% in the Netherlands [1]. However, lactose-free dairy has currently a wide and growing health appeal to all consumers and in countries where most people are lactose tolerant. Lactose-free dairy products can provide essential nutrients present in milk to people that are lactose intolerant. There are a number of dairy products that contain very little or no lactose, and these are generally well-tolerated by people that are intolerant to lactose. For some cheeses, e.g., Gouda, the production process includes a curd washing step to lower the lactose content [2]. Such cheeses already have a relatively low lactose content, even without ripening. However, for many other cheeses, the lactose content is only lowered due to the action of lactic acid bacteria during ripening. Hence, in general, aged, hard cheeses such as Parmesan, Cheddar or Swiss cheeses will have a very low lactose concentration. Young/fresh cheeses may, however, still contain sufficient lactose to result in a reaction among lactose-intolerant people, depending on the amount that is consumed [3]. Another dairy product that is low in lactose is butter. During butter production, most of the water-soluble components in milk, including lactose, are removed, thereby reducing the lactose content in butter to <0.1%.

However, many fresh dairy products do contain an appreciable amount of lactose [3]. Besides milk and milk drinks, these include fermented milk products like yoghurt, sour cream, crème fraiche, kefir etc.; ice cream; and other dairy desserts like dulce de leche, (whipped) dairy cream and sweetened condensed milk. Also, products made from whey (e.g., whey protein concentrates (WPC), whey protein isolates (WPI) and whey permeate) or milk powders will contain lactose. Outside the direct dairy space, many food products are, therefore, also not lactose free, like chocolate, coffee drinks and many baked products. 

For lactose intolerant people, it is nowadays not necessary to completely avoid the indulgence and nutritional value of dairy products [4]. There are very good solutions that rely on the hydrolysis of lactose into glucose and galactose with the enzyme lactase. These monosaccharides are readily adsorbed in the small intestine and prevent the occurrence of lactose intolerance symptoms. Two different type of lactases are currently commercially available: neutral lactases and acid lactases [5]. Neutral lactases are mainly used in the production of lactose-free dairy products on an industrial scale, although in some countries, this enzyme is also offered to consumers for treating milk at home. Acid lactase is available to consumers as a nutritional supplement to be taken together with regular dairy products and works by splitting lactose in the stomach. 

This review focusses on the former application: the industrial production of lactose-free dairy products using neutral lactases. We will touch upon the market developments, the industrial production processes and the nutritional consequences of the consumption of lactose-free dairy products. 

## 2. Market Developments of Lactose-Free Dairy

**Figure 1:**
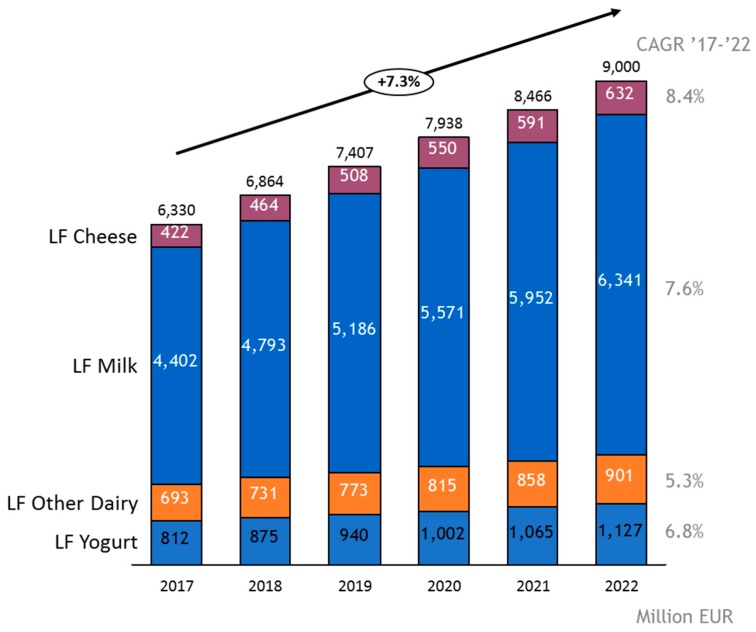
A compounded annual growth rate (CAGR) estimate of the lactose-free dairy market segment over the period 2017–2022. The total yearly turnover in M€ is indicated. LF: lactose-free. The figure was created from data of the Euromonitor analysis [6].

The lactose-free dairy market is the fastest growing segment in the dairy industry. Lactose-free dairy is expected to reach a €9 billion turnover by 2022 and continues to outgrow overall dairy (7.3% vs. 2.3%) (data extracted from a Euromonitor analysis [6]). Potable milk is the largest category of lactose-free dairy, represents two-thirds of the market and drives the absolute growth of the category (Figure 1). The second category is lactose-free yogurt, which will reach a €1 billion turnover by 2020. Lactose-free cheese is expected to grow fastest (8.4%) over the forecast period. Western Europe is the biggest and fastest growing lactose-free market, followed by Latin America.

The lactose-free segment drives the sales growth in the dairy industry, as demonstrated by the number of new product launches which have significantly increased between 2012 and 2016 (based on an analysis of the Mintel database [7]). Globally, the lactose-free claim is no longer only used for niche products; it is, in fact, among the top health claims in new milk product launches [7].

The wide availability of lactose-free products is encouraging consumers to make lactose-free a preferred choice for dairy, driven by the increasingly wide product range. Product diversity is one of the major shifts we are noticing in recent years. Lactose-free is appearing in an ever-wider range of dairy categories. Shelf-stable lactose-free dairy product sales increased only marginally, driven by launches in UHT (ultra-heat treated) lactose-free milk. This was traditionally the only lactose-free option to be found in most countries. However, the increasing number of launches in the chilled section is indicating the move to mainstream where it is easier for consumers to find it—often next to plant-based alternatives. Additionally, because of the excellent quality of the current lactose-free dairy products, households are often completely switching to lactose-free dairy when a single member is lactose intolerant, stimulating sales in this segment.

The enzyme used for the production of lactose-free dairy products has traditionally been the neutral β-galactosidase derived from the dairy yeast *Kluyveromyces lactis* (and its close relatives *Saccharomyces lactis*, *K. marxianus* or *K. fragilis*) [5]. This classical enzyme is produced by one European and three Japanese companies: DSM Food Specialties (Heerlen, the Netherlands) (exclusively sold under the brand name Maxilact^®^), Godo (Japan), Amano (Japan) and Nagase (Japan). The Japanese companies market their products via second suppliers like Dupont (Wilmington, DE, USA) (Godo YNL2^®^), Chr. Hansen (Øresund, Denmark) (Halactase^®^) and Novozymes (Bagsværd, Denmark) (Lactozyme^®^ Pure) and by many other suppliers under different brand names. All commercial enzymes from *K. lactis* have basically the same performance in lactose hydrolysis, but different product strengths and purity grades are available (see also Section 3). Other commercial β-galactosidases from, for example, *Bacillus circulans* or *Aspergillus oryzae* are less suitable for producing lactose-free products in most dairy matrices because of the different pH or temperature optimums [5] and are mainly sold for either galacto-oligosaccharide (GOS) production or as a nutritional enzyme. A recent market development is the supply of β-galactosidases produced with genetically modified production hosts. Saphera^®^ is sold by Novozymes (but also as Nola-Fit^®^ by Chr. Hansen) and is a truncated version of a *Bifidobacterium* lactase produced with a *Bacillus* host strain. Maxilact^®^ Smart is produced and sold by DSM Food Specialties, and is a self-cloned version of the regular *K. lactis* lactase with a higher specific activity. It will be interesting to see if these new products will be accepted in the market and what their added value for the dairy producer will be. All commercial lactase enzymes used for the production of lactose-free milk are available as stabilized liquids and are certified Kosher/Halal. 

## 3. Production of Lactose-Free Dairy

In this section, we will describe the different processes that are used in the dairy industry for the production of lactose-free products. Also, we will touch upon the effects that lactose hydrolysis may have on the processing and quality aspects when producing lactose-free dairy. The different lactose-reduced dairy products are mentioned here in order of their economic relevance.

### 3.1. Milk

Potable cow’s milk for dairy intolerant people is available in many countries in different forms. Although most people with lactose intolerance have few symptoms with a lactose dosage of <12 gram per meal [4], the general trend in this industry is to reduce the lactose content as low as possible. There is no global consensus on the regulatory requirements for lactose-free claims. While in the past most dairy producers regarded lactose reduction to 0.5% or 0.1% as sufficient, the current requirement in some countries is even a reduction to <0.01% before the milk can be called lactose-free. A half-liter of lactose-free milk will, therefore, have less than 50 mg of lactose, much less than would be required from a nutritional point of view. Obtaining such low amounts of lactose in milk production requires special attention not only to the processing of the milk and to the dosage and effectiveness of the enzyme used in this process but also to sensitive analytical methods to determine such low amounts of lactose [8]. 

In general, the majority of potable milk in Northern European countries, North America and Australia is pasteurized and stored under cooled conditions for a couple of weeks. In Southern European countries, South America and Asia most milk is UHT sterilized and stored at ambient temperatures up to 9 months. Curiously, besides the consumer preference for the flavor of either pasteurized or UHT sterilized milk, the preference for sweetness in milk roughly divides between these lines: the extra sweetness generated in milk by lactose hydrolysis is especially appreciated in Latin and Asian countries but disliked in traditional milk-drinking areas like Northern Europe and North America. 

Currently, two processes (batch and aseptic) are in use to produce lactose-free milk [9,10], and both these processes use soluble lactase enzyme. Processes relying on an immobilized enzyme have been suggested in the scientific literature a long time ago and even trialed on a pilot scale [11]. However, the immobilization of lactase was not used in industrial practice for lactose-free milk production until today due to problems with the microbial stability of the final product. The recycling of the immobilized enzyme in an industrial setting is therefore limited, making the procedure less cost-effective and more prone to quality defects than hydrolysis with a soluble enzyme.

#### 3.1.1. Batch Process (Pre-Hydrolysis)

**Figure 2:**
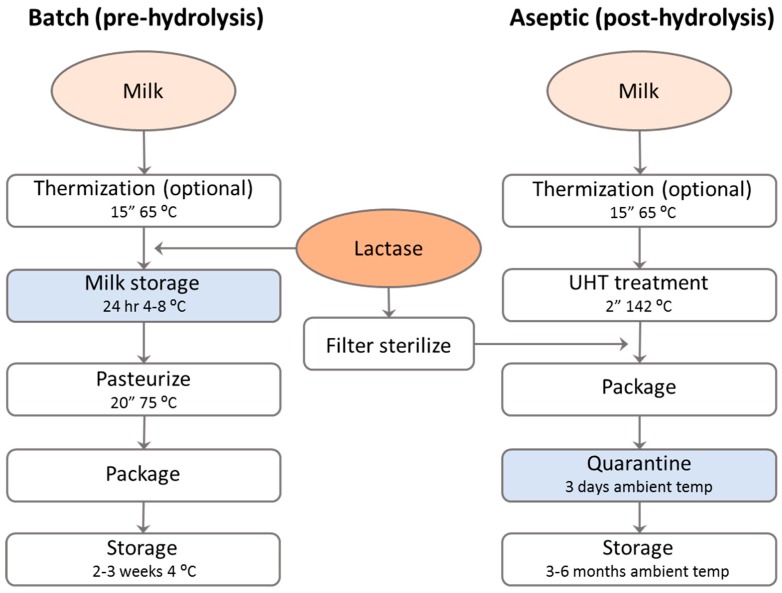
A schematic representation of the batch (left) and aseptic (right) processes that are used to produce lactose-free milk. The process conditions may vary from one factory to another, and additional process steps (like homogenization and standardization) are commonly included before the heat treatment. The batch process may include UHT treatment. The process step where lactose hydrolysis takes place is indicated in blue.

In the batch process, a neutral lactase sample is added to a tank of raw or thermized milk and, commonly, incubated for approx. 24 h under slow stirring to prevent creaming. Since the milk at this stage is not sterile yet, this process has to be performed at cooled conditions (normally 4–8 °C) to prevent microbial growth. After this incubation, the milk is pasteurized, homogenized and packaged (Figure 2). Also, some producers of UHT milk use the batch process, although the aseptic process has become much more popular for this market segment in recent years. Since the enzyme is inactivated during the pasteurization/sterilization of the milk, no residual enzyme activity remains in the final product when the milk is produced via the batch process, which is an advantage of the regulation and labelling in some countries. 

A number of aspects are important to consider when one uses the batch process for making lactose-free milk:

- The dosage of the enzyme should be sufficient to reach the required boundary for lactose-free during the limited time and low temperature of the incubation. Therefore, the enzyme dosage is relatively high. Enzymes available for this process are selected for their relatively high activity at neutral pH and low temperature. The process control is high since the lactose conversion may be measured in the tank and since the enzyme dosage or incubation time may be adapted during the process.
- The batch incubation requires the occupation of a tank in the factory and the holdup of the milk for a day. The process is, therefore, discontinuous, which may pose a problem for some factories, especially when the productivity is high. A lactase with a higher specific activity under these conditions (like Maxilact^®^ Smart) may help shorten the production time and, hence, may increase the throughput of the factory. Since the pasteurization of the milk is postponed for a day, the milk quality should be impeccable to prevent microbial spoilage.
- The milk product produced with the batch process is relatively insensitive for possible side activities in the enzyme preparation. This is due to the limited time of storage of the milk at refrigerated conditions and to the pasteurization/sterilization after the enzyme incubation, inactivating most enzymatic activities. Although in the past some lactase preparations showed proteolytic side activities [12], these problems seem to be over and very few complaints occur for lactose-free milk produced with the batch process.
- Since lactose hydrolysis leads to a doubling of the sweetness of milk, processes were developed to remove part of the lactose using chromatography or (ultra and nano) filtration techniques combined with the hydrolysis of the remaining lactose so an exact sweetness is regenerated [13,14]. The resulting lactose-free milk produced with this process is of excellent quality, and the taste is almost identical to regular milk. This feature is especially appreciated by the pasteurized milk drinkers in Northern Europe and North America and is, therefore, frequently used in conjunction with the batch process.

#### 3.1.2. Aseptic Process (Post-Hydrolysis)

In the aseptic process, the milk is first sterilized using the UHT procedure, after which a sterile lactase preparation is injected into the milk just before packaging [15]. The lactose conversion in the milk will take place in the milk package (Figure 2). Since UHT milk is often kept in quarantine for approx. 3 days at ambient temperature, there is sufficient time for complete hydrolysis before the milk is shipped to the retailer. Since there is no quarantine period for pasteurized milk, the aseptic process is not used for this type of lactose-free milk. There are, in essence, two different procedures for obtaining a sterile lactase. In the first procedure, the lactase enzyme is presterilized by the manufacturer of the enzyme, and special sterile dosing equipment is required for the sterile injection (e.g., the Tetrapak (Lund, Sweden) Flexdos^®^ system). In the second procedure, the unsterile enzyme is filter-sterilized just before addition to the sterile milk at the dairy factory (via, for example, the Tetrapak Aldose^®^ system).

A number of aspects are important to consider when one uses the aseptic process for making lactose-free milk:

- The dosage of the enzyme can be much lower compared to the batch process, since both the incubation time and temperature are higher. Process control is, however, absent since the enzyme is only active in the final milk package. E.g., the storage temperature in un-thermostated warehouses may deviate from summer to winter, and the dairy producer should take these aspects into account when dosing the enzyme.
- The aseptic process requires special equipment and consumable costs, and especially for the in-factory filtration, it requires highly skilled operators to prevent microbial contamination of the milk during lactase injection. However, the process can be operated full-continuous when organized properly, and that is a major advantage for factories that require a high throughput.
- The aseptic process for making lactose-free UHT milk could only be fully developed after major improvements in the quality of the lactase enzymes. Besides the removal of proteolytic side activity, it was also found that arylsulfatase side activity in the lactose preparation may lead to severe medicinal off-flavors during storage due to *p*-cresol formation from sulphonated-cresol that is naturally present in the milk [16]. A producer of lactose-free UHT milk should consider using only the highest quality lactases for this process to prevent problems during shelf life. Arylsulfatase-free lactases (like all Maxilact^®^ products) are currently commercially available.
- Lactose hydrolysis in milk leads to an increased presence of monosaccharides, and therefore, the Maillard reaction is more efficient. Limited proteolysis by proteases present in the milk or originating from the lactase preparation may enhance the reaction. This results in the increased formation of off-flavors, in the browning of lactose-free milk when compared to regular milk and in a reduced nutritional value when stored at increased temperatures [10,17,18]. The increased Maillard reaction is probably the most important determinant of the reduced shelf life of lactose-free UHT milk compared to regular UHT milk. Although it has been suggested in the past that lactose-free UHT milk production using the batch process may lead to even more browning compared to milk produced via the aseptic process [19], recent data show that the storage conditions (temperature) and choice of the lactase are much more relevant for determining shelf life [10]. Excellent shelf life was found for lactose-free UHT milk produced with the batch process, and milk browning during storage is, therefore, largely independent of the production process that is used.

### 3.2. Fermented Milk Products

The presence of a substantial amount of lactose in most fermented milk products, like 30–40 gram/kg in yoghurt [3], would suggest that lactose-intolerant people will have a problem with these dairy products. However, this seems to be much less the case than expected from the amount of lactose that is consumed. There have been two different theories put forward to explain this phenomenon (reviewed by References [20,21]).

- It has been suggested that the lactic acid bacteria present in yoghurt will survive the stomach, and the lactase enzyme present in these bacteria aids the digestion of (or part of) the lactose in the small intestine. The monosaccharides are both consumed by the bacteria and taken up in the small intestine, so lactose intolerance symptoms are reduced. Some dairy companies claim to produce a yoghurt containing special cultures that have this effect. This hypothesis will only hold if the yoghurt bacteria and their intracellular lactase enzyme will survive the stomach. Indeed, the pasteurization of yoghurt seems to worsen lactose intolerance symptoms.
- A second explanation that was put forward suggests that the lactose in yoghurt is better digested due to the decreased transit time of a viscous yoghurt meal compared to liquid milk. Due to this, any residual lactase in the small intestine will have more time to digest lactose and, thereby, reduces intolerance symptoms. It has indeed been found that having a meal together with a glass of milk will reduce symptoms, suggesting that transit time may play a role in lactose digestion.

**Figure 3:**
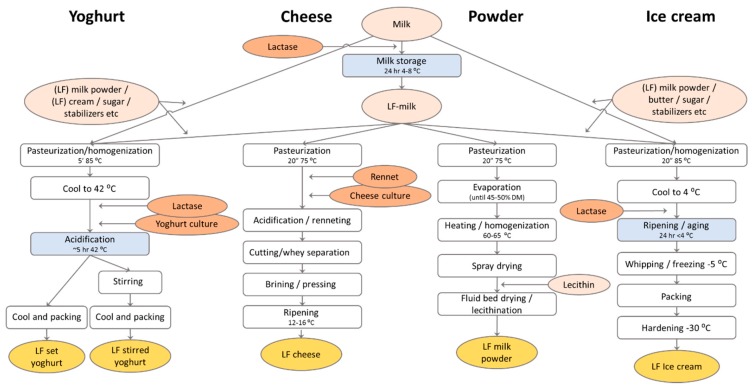
A flow scheme for the production of different lactose-free (LF) dairy products: The process conditions may vary from one factory to another, and additional process steps and additions are commonly included. All products can be made from LF-dairy ingredients, but yoghurt and ice cream may also be treated with lactase in the process. The process steps where lactose hydrolysis can take place are indicated in blue.

Regardless of which of these effects play the major role in tolerating fermented milk products by lactose intolerant people, the most reliable remedy seems to be the complete enzymatic digestion of lactose in yoghurt. This can be done by incubating the milk with lactase before pasteurization (familiar to the batch process for milk) or adding the lactase together with the culture after the pasteurization of the milk (Figure 3). Most yoghurt producers opt for the latter, co-hydrolysis, approach since predigestion seems to inhibit the activity of some yoghurt cultures (see, for example, Reference [22]), probably due to the switch from lactose to glucose as a main carbon source or to the increased osmotic pressure in lactose-hydrolyzed milk.

When the (neutral) lactase is added to the milk at the same time as the yoghurt culture, only a limited time is left for lactose digestion. Most neutral lactases are completely inactivated at a pH < 5.5 [5], which is reached after 2.5–3 hours of incubation in a regular yoghurt making process. Hence, the lactase dosage must be relatively high to obtain a lactose-free status. Acid stable lactases are presently on the market, and these may reduce the total enzyme dosage. However, since these enzymes are not inactivated by the low pH in the final product, the addition has to be labeled on the yoghurt package in some countries.

A major advantage of using lactases in the production of yoghurt is the increase in sweetness due to the splitting of lactose [23,24]. Hence, the total added sugar can be reduced by 1.5–2 g/100 g without changing the flavor profile. The enzyme addition can be reduced for this application since the final product does not have to be lactose-free, but a slightly higher residual lactose is allowed without noticeable difference in sweetness. Since *K. lactis* lactase is inactivated in the yoghurt process due to the pH drop, the labelling of the enzyme on the yoghurt package is often not required. Hence, many yoghurt manufacturers use the enzyme as a label-friendly solution to reduce the sugar addition.

Another advantage of the digestion of lactose in yoghurt is that the post-acidification during shelf life can be reduced when specific yoghurt cultures are used [25]. Apparently, some yoghurt bacteria are less active in the absence of lactose or have difficulties in switching from one carbon source (lactose) to another (glucose), leading to a better sensory stability of the product. 

Invertase activity was present as a side activity in many commercial lactases in the past. Invertase digests sucrose into glucose and fructose and, thereby, influences the sweetness perception in a fermented milk product that contains added sucrose. This problem was recognized and has led to the development of special lactases without invertase activity, such as Maxilact^®^ LGi. 

Other relevant side activities may disturb the texture when, for example, starch or pectin has been used in the manufacturing of the fermented milk product. Amylase (e.g., α-amylase or glucoamylase) and pectinase (e.g., endo-galactanase, pectin lyase or polygalacturonase) side activities, often present in bacterial or fungal enzyme preparations, may affect the texture of these fortified yoghurts during production or storage, and lactases lacking such side activities are, therefore, preferred. Fortunately, most neutral lactases are made with the dairy yeast *Kluyveromyces lactis* that lacks such activities. No significant textural differences were detected when starch-based desserts were produced using such lactose-free milk [26].

### 3.3. Other Dairy Products

Many other dairy products are made lactose-free using an enzymatic treatment. Every application has its own pitfalls when lactose is converted into monosaccharides, so it is important to realize that, often, the process or the recipe has to be adapted to obtain the optimal result.

- Flavored milk is made with a process that is familiar to the milk process (Figure 2). However, the generation of the extra sweetness by the lactase treatment is an advantage since it will allow a reduction of sugar addition, similar to the situation in most yoghurts [24]. Flavored lactose-free milk also has much less problems with Maillard-related off-flavors and browning than regular lactose-free UHT milk, since the product often has a strong flavor and color by itself. For some flavored milks, like highly sugared chocolate milk, lactose hydrolysis may be insufficient to fully replace all sugar addition [27], so the addition of additional sweeteners may still be required.
- Dairy powders can be produced from milk or whey that is made lactose-free via the batch process (Figure 3). A major problem is the presence of a high concentration of monosaccharides in the treated milk, leading to a drop in the glass-transition temperature. Hence, this product will lead to the fouling of the spray dryer when the drying conditions are not adapted [28]. The much milder spray drying conditions lead to dramatic decreases in the productivity of the drying process and to increased costs. Additionally, the lactose-free milk (or whey) powder is highly hygroscopic, leading to caking during storage when not packaged with extra caution. Due to these challenging technical problems, lactose-free dairy powders is still a small market in contrast to regular milk powders.
- Cheese can be made lactose-free by incubating the cheese milk with lactase before renneting (Figure 3). This is mostly useful for young, fresh cheeses that are known to contain a significant amount of lactose, and lactases are currently used for this purpose. In ripened cheeses, all lactose will have been consumed by the lactic acid bacteria, so no lactase incubation is required. In contrast to the situation in yoghurt, the treatment of cheese milk with lactase is in the older literature often mentioned to stimulate the acidification during cheese making. Also, the lactase addition is mentioned to increase cheese flavor formation during ripening. However, it is not entirely clear if these effects are due to the stimulation of the cheese microbial flora due to the hydrolysis of the lactose or to residual proteolytic activity present in the lactase preparations that were commercially available in the past. The release of amino acids from milk proteins may have stimulated the cheese culture activity in these experiments. The latter explanation seems to be the most likely (see, for example, the discussion on this subject in Reference [29]).
- Ice cream can also be made lactose-free by either using lactose-free milk and powders in the ice cream mix [30] or by adding the lactase enzyme after pasteurization and incubation during the aging period before freezing (Figure 3). Because of the increase in monosaccharide content after lactose hydrolysis, the freezing point of the ice cream mix will decrease. This will lead to a softer ice cream at the same temperature. Although for some frozen desserts this may be an advantage because of the “soft scoop”, it will also lead to faster melting. Since the sweetness increases due to lactose hydrolysis, the ice cream maker can, however, decide to decrease sugar addition and, thereby, increase the melting temperature again. Since such changes in the recipe will decrease total solids in the mix, additional measures may be required to comply to the local regulation for ice cream. Lactase treatment is also used in ice cream to prevent lactose crystallization. Especially when whey powder or WPC is used in the ice cream mix, the amount of lactose may be high enough to form crystals during freezing. This leads to a sensory defect that is called “sandiness”. Lactase treatment can prevent the formation of sandiness in ice cream by splitting lactose into glucose and galactose, which are more soluble at low temperature. It was found that lactose hydrolysis increases the apparent viscosity of the ice cream mix, decreases the freezing point, increases the sweetness to allow a 25% reduction in sugar addition, decreases sandiness, and improves the overall acceptability of ice cream [30].
- Dulce de Leche is produced by heating sugared concentrated milk until caramelization occurs due to Maillard reactions. Lactase treatment of the milk will not only make the final product lactose-free but also stimulate the Maillard reaction due to the release of galactose. Hence, treatment with lactase may enhance the flavor and color formation in the production process for Dulce de Leche. Additionally, like in ice cream, lactose hydrolysis prevents the occurrence of sandiness when the final product is stored refrigerated.
- For many dairies, the cheese whey is a valuable by-product that is fractionated into proteins, lactose and milk salts and sold as WPC, WPI and different grades of lactose. However, not all dairies have the capabilities or facilities to produce these products. The sweet dairy flavor of liquid whey or whey permeate may also be upgraded by digesting the lactose with a neutral lactase, either in free form, immobilized or as cell suspension, before concentrating it into a syrup or powder [31]. Such lactose-free products may be used as sweetener in, for example, ice cream, confectionary or bakery applications. An additional advantage of hydrolyzing the lactose in whey or whey permeates is that a microbially stable syrup may be formed without crystallization problems, thereby saving on drying costs. Depending on the pH of the substrate, either neutral or acid lactase may be used for the conversion [31].

Besides these examples of lactose-free dairy products where lactase-treatment may have additional advantages, many other lactose-free dairy products like desserts, creams, etc. are nowadays being produced and aid to expand the lactose-free product portfolio. 

## 4. Health Aspects of Lactose-Free Dairy 

### 4.1. Nutritional Difference between Lactose-Free Dairy and Normal Dairy

Lactose-free dairy may confer benefits to lactose intolerant people, allowing them to enjoy the taste of dairy without the uncomfortable intestinal symptoms from the ingestion of lactose. In addition, lactose-free dairy also has a growing health appeal to lactose tolerant people. In lactose-free dairy, the lactose is predigested into glucose and galactose. Consequently, the lactose content may be very low (<0.1 g/L), but the glucose and galactose content of lactose-free milk will be approx. 25 g/L. As mentioned above, the glucose and galactose in lactose-free dairy are sweeter than lactose, enabling a reduction of the added sugar in dairy products by up to 10–15 g/kg, thereby reducing calorie addition [24]. Besides the advantages of reduced lactose intake for lactose intolerant people, lactose-free dairy is not likely to have different nutritional effects on the human body as compared to normal dairy. When predigested lactose is consumed, the glucose and galactose will also be absorbed in the small intestine like will be the case for the digestion products glucose and galactose when intact lactose is consumed by lactose tolerant dairy consumers. No difference in gastric emptying was found in rats when comparing lactose versus glucose and galactose consumption [32]. A study in calves found no difference in the glycemic responses between milk and lactose-free milk [33]. The absence of differences in glycemic response between milk and lactose-free milk was confirmed by a study on healthy subjects (internal unpublished data). Also, no difference was observed in the glycemic response of diabetes patients when consuming lactose compared to the lactose digestion products glucose and galactose [34].

### 4.2. Potential Health and Economic Impact of Lactose-Free Dairy vs. Dairy Avoidance

The National Institute of Health (NIH) concluded that the vast majority of lactose mal-absorbers will tolerate up to 12 g of lactose per serving and that smaller amounts of lactose will generally not cause major problems [4]. Still, most subjects with self-diagnosed or physician-diagnosed lactose intolerance will try to avoid all lactose-containing products. However, in its Updated Consensus Statement, the National Medical Association reports that lactose free dairy products are the most ideal substitute for regular dairy products among individuals with lactose intolerance. In addition, evidence indicates that children prefer lactose-free cow’s milk over soy beverages [35].

Table 1 shows the nutritional composition of cow’s milk [36]. Excluding all dairy products from the diet can lead to nutrient deficiencies because these foods are a major source of nutrients, notably choline, phosphorus, calcium, riboflavin and vitamin B12 and A (Table 1). In the US and Canada, milk is mandatorily fortified and is an important source of vitamin D. “Dairy alternatives” from soy, rice or almonds are often consumed as a substitute for cow’s milk or yoghurt, constituting a nutritious alternative if fortified with vitamin A, vitamin B12 and calcium. One study found that calcium bioavailability from fortified soy milk was only 75% of the efficiency of calcium from cow milk [37]. Consequently, most nondairy lactose-free products are fortified with at least 20% more than the recommended intake for calcium. 

Alternatives that do not exclude cow’s milk include lactose-free milk products, yogurt containing lactic acid bacteria and cow’s milk combined with an intake of lactase supplements. Lactose-free dairy products and lactase supplements may have an advantage over excluding dairy products from the diet in that they do not reduce dietary intake and its essential nutrients. There is, however, still controversy surrounding the role of lactose in enhancing calcium and divalent mineral bioavailability. Lactose has been recognized as an enhancer of calcium absorption in mammals; in animal studies, lactose was found to enhance calcium absorption, but in humans, this effect is still debated [38]. It was suggested that conflicting results are due to differences in the control sugars, where the component sugars glucose or galactose gave comparable calcium absorption as lactose, while other sugars did not. 

Little information is available about the food choices of lactose intolerant people, but in the US, 75% of people with lactose intolerance avoid dairy with over half of them worrying about the long-term risks to their health due to this dietary restriction [39]. Dairy avoiders have different reasons to avoid it; in a survey in the US, 61% of respondents indicated an avoidance of dairy for intolerance or allergy/sensitivity, while the remainder avoided dairy for other reasons [40]. A survey in Canada showed that despite the higher use of calcium and vitamin D supplements, lactose intolerants had lower calcium and vitamin D intake from the combination of dairy products, alternatives and supplements [41].

Some studies are available, reporting on the nutrient intake among lactose-avoiding subjects. Results generally show that lactose intolerant compared to tolerant people consume lower amounts of calcium with average amounts below the RDA (recommended dietary allowance) of 1000 mg/d (577 [43], 692 [44], 388 [45], 510 [46] and 739 [41] mg/d).

Observational studies have shown that the avoidance of dairy foods was associated with adverse health outcomes, including poor bone health [47,48], higher blood pressure [49] and a higher risk of developing diabetes [50]. The EPIC-Oxford cohort study with 34,696 British people showed that vegans were at 30% higher risk for bone fractures than omnivores and vegetarians (who do consume milk, yoghurt, cheese and eggs), which was attributable to lower calcium intake [51]. However, the possibility of important confounding bias in observational studies cannot be fully excluded. A recent randomized controlled intervention study investigated the effect of 18 months dairy consumption in healthy boys and girls on bone mass. No effects were found on any of the bone parameters except for tibial bone mineral content gain, which was greater in the group receiving dairy [52]. However, in a longer study in teenage girls receiving dairy for two years, dairy significantly increased bone mineral density at the trochanter, femoral neck and lumbar spine [53]. The effect of calcium and vitamin D intake on bone has been well-established. Vitamin D either in combination with calcium or not reduces the likelihood of hip fractures and other types of fracture in postmenopausal women or men aged over 65 years [54]. As calcium and vitamin D intakes in lactose intolerants are generally below the recommended intakes, it is likely that this may lead to a decreased bone density, especially in older men and women. 

Moreover, evidence exists that dairy consumption is inversely related to blood pressure [55], which may be due to the presence of calcium, vitamin D and bioactive peptides in dairy.

When unaware, lactose intolerance can cause symptoms very similar to Irritable Bowel Syndrome (IBS), which may impair health-related quality of life. Among dairy-avoiding subjects, worry about an accidental lactose intake and a modification of diet may also affect the quality of life to some extent. 

Two studies reported on the impact of lactose intolerance on the quality of life. In one study, the self-perception of intolerance was associated with lower health-related quality of life scores (median: 60 vs. 70, *p* < 0.01) [56]. In contrast, another survey did not find a negative impact of being intolerant to lactose on this score [57].

One study estimated the potential impact of improved dairy consumption on reducing the disease burden in the Netherlands, France and Sweden. The impact of increasing calcium daily intake by 650 mg (equaling approximately 200 mL milk, 125 mL yoghurt and 30 g cheese) to reach the recommended intake has been modelled. For instance, in France, increasing dairy intake could prevent 2023 hip fractures per year, 6263 Disability-Adjusted Life Years (DALYs) per year and 129 million euro per year [58]. The impact of increasing dairy intake in the Netherlands was lower as dairy intake is already relatively high. Besides the individual health benefits of dairy consumption, the economic benefits for society are evident, suggesting that the promotion of the consumption of lactose-free dairy products in countries with a high prevalence of lactose intolerance makes sense. 

## 5. Conclusions and Future Scope 

Lactose-free dairy products are becoming more mainstream and provide excellent opportunities for lactose intolerant people to benefit from the broad palette of different nutritious and delicious products made from milk. Consumer awareness of the nutritional relevance of dairy products combined with the promotion of the many different lactose-free dairy possibilities and their benefits (like sugar reduction and prevention of sandiness) may increase the market penetration of these products even further. For individual dairy makers, the lactose-free segment seems to be a highly interesting, profitable and expanding market where innovative new products can flourish. For the dairy industry as a whole, lactose-free products may attract different consumers that were not well-served by the traditional dairy products, and hence, these products offer the possibility to expand the total market. In the future, we expect to see many different new product launches in this rapidly expanding segment of the dairy industry.

## Figures and Tables

**Table 1:** The nutritional composition of cow’s milk with 3.25% fat as the % of Daily Value (DV) per 100 g and per 244 g serving [42].

		per 100 g	% of DV per 244 g Cup
Macronutrients			
Energy	kcal	61	
Protein	g	3.2	15%
Total lipid (fat)	g	3.3	12%
Carbohydrate	g	4.8	4%
Choline	mg	34.9	15% ^1^
Minerals			
Calcium, Ca	mg	113	28%
Iron, Fe	mg	0.03	0%
Magnesium, Mg	mg	10	6%
Phosphorus, P	mg	84	20%
Potassium, K	mg	132	7%
Sodium, Na	mg	43	13%
Zinc, Zn	mg	0.37	6%
Vitamins			
Vitamin C	mg	0	0%
Thiamin	mg	0.046	7%
Riboflavin	mg	0.169	24%
Niacin	mg	0.089	1%
Vitamin B-6	mg	0.036	4%
Folate, DFE	µg	5	3%
Vitamin B12	µg	0.45	18%
Vitamin A, RAE	µg	46	8%
Vitamin E	mg	0.07	1%
Vitamin D	µg	0.1	1%
Vitamin K	µg	0.3	1%

^1^ Choline has no DV but has adequate intake.

## References

[1] Silanikove N., Leitner G., Merin U. (2015). The Interrelationships between Lactose Intolerance and the Modern Dairy Industry: Global Perspectives in Evolutional and Historical Backgrounds. Nutrients.

[2] Walstra P., Geurts T.J., Noomen A., Jellema A., van Boekel M.A.J.S. (1999). Dairy Technology: Principles of Milk Properties and Processes.

[3] The Really BIG List of Lactose Percentages. http://www.stevecarper.com/li/list_of_lactose_percentages.htm.

[4] Suchy F.J., Brannon P.M., Carpenter T.O., Fernandez J.R., Gilsanz V., Gould J.B., Hall K., Hui S.L., Lupton J., Mennella J. (2010). National Institutes of Health Consensus Development Conference: Lactose intolerance and health. Ann. Intern. Med..

[5] Dekker P.J.T. (2016). Enzymes Exogenous to Milk in Dairy Technology: β-D-Galactosidase. Reference Module in Food Sciences.

[6] Euromonitor Database. https://www.euromonitor.com/.

[7] Mintel Global New Product Database. http://www.mintel.com/global-new-products-database.

[8] Van Scheppingen W.B., van Hilten P.H., Vijverberg M.P., Duchateau A.L.L. (2017). Selective and sensitive determination of lactose in low-lactose dairy products with HPAEC-PAD. J. Chromatogr. B..

[9] Harju M. (2004). Chromatographic and enzymatic removal of lactose from milk. Bull. Int. Dairy Fed..

[10] Troise A.D., Bandini E., De Donno R., Meijer G., Trezzi M., Fogliano V. (2016). The quality of low lactose milk is affected by the side proteolytic activity of the lactase used in the production process. Food Res. Int..

[11] Finocchiaro T., Olson N.F., Richardson T. (1980). Use of immobilized lactase in milk systems. Adv. Biochem. Eng..

[12] Mittal S.B., Newell G., Hourigan J.A., Zadow J.G. (1991). The effect of protease contamination in lactase on the flavour of lactose-hydrolysed milks. Aust. J. Dairy Technol..

[13] Jelen P., Tossavainen O. (2003). Low lactose and lactose-free dairy products–prospects, technologies and applications. Aust. J. Dairy Technol..

[14] Harju M., Kallioinen H., Tossavainen O. (2012). Lactose hydrolysis and other conversions in dairy products: Technological aspects. Int. Dairy J..

[15] Dahlqvist A., Asp N.-G., Burvall A., Rausing H. (1977). Hydrolysis of lactose in milk and whey with minute amounts of lactase. J. Dairy Res..

[16] De Swaaf M.P.M., van Dijk A.A., Edens L., Dekker P.J.T. (2006). Enzyme Preparation Yielding A Clean Taste.

[17] Jansson T., Jensen H.B., Sundekilde U.K., Clausen M.R., Eggers N., Larsen L.B., Ray C., Andersen H.J., Bertram H.C. (2014). Chemical and proteolysis-derived changes during long-term storage of lactose-hydrolyzed ultrahigh-temperature (UHT) milk. J. Agric. Food Chem..

[18] Evangelisti F., Calcagno C., Nardi S., Zunin P. (1999). Deterioration of protein fraction by Maillard reaction in dietetic milks. J. Dairy Res..

[19] Mendoza M.R., Olano A., Villamiel M. (2005). Chemical indicators of heat treatment in fortified and special milks. J. Agric. Food Chem..

[20] Kies A.K., Sadler M.J. (2014). Authorised EU health claims related to the management of lactose intolerance: Reduced lactose content, dietary lactase supplements and live yoghurt cultures. Foods, Nutrients and Food Ingredients with Authorised EU Health Claims.

[21] Savaiano D.A. (2014). Lactose digestion from yogurt: Mechanism and relevance. Am. J. Clin. Nutr..

[22] Kárnyáczki Z., Csanádi J. (2017). Texture profile properties, sensory evaluation, and susceptibility to syneresis of yoghurt prepared from lactose-free milk. Acta Aliment..

[23] Adhikari K., Dooley L.M., Chambers E., Bhumiratana N. (2010). Sensory characteristics of commercial lactose-free milks manufactured in the United States. Lebensm. Wiss. Technol..

[24] McCain H.R., Kaliappan S., Drake M.A. (2018). Sugar reduction in dairy products. J. Dairy Sci..

[25] Garrigues C., Gilleladen C., Curic-Bawden M., Janzen T., Birkelund M., Buchhorn G.L., Soerensen K.I., Christensen N., Svane C., Riis S. (2015). Method of Producing A Fermented Milk Product with Improved Control of Post-Acidification.

[26] Sahin A., Hamamci H., Garayev S. (2016). Rheological properties of lactose-free dairy desserts. Food Sci. Technol. Int..

[27] Li X.E., Lopetcharat K., Qiu Y., Drake M.A. (2015). Sugar reduction of skim chocolate milk and viability of alternative sweetening through lactose hydrolysis. J. Dairy Sci..

[28] Ferreira Torres J.K., Stephani R., Miranda-Tavares G., de Carvalho A.F., Golin Bueno Costa R., Rocha de Almeida C.E., Ramos Almeida M., Cappa de Oliveira L.F., Schuck P., Tuler Perrone I. (2017). Technological aspects of lactose-hydrolyzed milk powder. Food Res. Int..

[29] Marschke R.J., Nickerson D.E.J., Jarrett W.D., Dulley J.R. (1980). A cause of increased proteolysis in cheddar cheese manufactured from milk containing added Maxilact. Aust. J. Dairy Technol..

[30] Abbasi S., Saeedabadian A. (2015). Influences of lactose hydrolysis of milk and sugar reduction on some physical properties of ice cream. J. Food Sci..

[31] Panesar P.S., Kennedy J.F. (2012). Biotechnological approaches for the value addition of whey. Crit. Rev. Biotechnol..

[32] da-Costa-Pinto E.A., Collares E.F. (1997). Chronic lactose intake modifies the gastric emptying of monosaccharides but not of disaccharides in weanling rats. Braz. J. Med. Biol. Res..

[33] Gutzwiller A. (2000). Glucose and galactose absorption after ingestion of milk containing hydrolysed lactose in calves with diarrhoea. J. Vet. Med. A Physiol. Pathol. Clin. Med..

[34] Ercan N., Nuttall F.Q., Gannon M.C., Redmon J.B., Sheridan K.J. (1993). Effects of glucose, galactose, and lactose ingestion on the plasma glucose and insulin response in persons with non-insulin-dependent diabetes mellitus. Metabolism.

[35] Bailey R.K., Fileti C.P., Keith J., Tropez-Sims S., Price W., Allison-Ottey S.D. (2013). Lactose intolerance and health disparities among African Americans and Hispanic Americans: An updated consensus statement. J. Natl. Med. Assoc..

[36] United States Department of Agriculture Food (USDA) USDA Food Composition Databases. https://ndb.nal.usda.gov/.

[37] Heaney R.P., Dowell M.S., Rafferty K., Bierman J. (2000). Bioavailability of the calcium in fortified soy imitation milk, with some observations on method. Am. J. Clin. Nutr..

[38] Kwak H.-S., Lee W.-J., Lee M.-R. (2012). Revisiting lactose as an enhancer of calcium absorption. Int. Dairy J..

[39] Survey: 75% of People With Lactose Intolerance Avoid Dairy Foods. http://www.marketwired.com/press-release/survey-75-of-people-with-lactose-intolerance-avoid-dairy-foods-1751282.htm.

[40] Half of Dairy Consumers in the U.S. Also Use Dairy Alternatives, New Research Out of Cargill Shows. http://www.nutritionaloutlook.com/food-beverage/half-dairy-consumers-us-also-use-dairy-alternatives-new-research-out-cargill-shows.

[41] Barr S.I. (2013). Perceived lactose intolerance in adult Canadians: A national survey. Appl. Physiol. Nutr. Metab..

[42] USDA Food Composition Databases. https://ndb.nal.usda.gov/ndb/search/list?home=true.

[43] Nicklas T.A., Qu H., Hughes S.O., He M., Wagner S.E., Foushee H.R., Shewchuk R.M. (2011). Self-perceived lactose intolerance results in lower intakes of calcium and dairy foods and is associated with hypertension and diabetes in adults. Am. J. Clin. Nutr..

[44] Segal E., Dvorkin L., Lavy A., Rozen G.S., Yaniv I., Raz B., Tamir A., Ish-Shalom S. (2003). Bone density in axial and appendicular skeleton in patients with lactose intolerance: Influence of calcium intake and vitamin D status. J. Am. Coll. Nutr..

[45] Buchowski M.S., Semenya J., Johnson A.O. (2002). Dietary calcium intake in lactose maldigesting intolerant and tolerant African-American women. J. Am. Coll. Nutr..

[46] Corazza G.R., Benati G., Di Sario A., Tarozzi C., Strocchi A., Passeri M., Gasbarrini G. (1995). Lactose intolerance and bone mass in postmenopausal Italian women. Br. J. Nutr..

[47] Heaney R.P. (2000). Calcium, dairy products and osteoporosis. J. Am. Coll. Nutr..

[48] Huncharek M., Muscat J., Kupelnick B. (2008). Impact of dairy products and dietary calcium on bone-mineral content in children: Results of a meta-analysis. Bone.

[49] Miller G.D., DiRienzo D.D., Reusser M.E., McCarron D.A. (2000). Benefits of dairy product consumption on blood pressure in humans: A summary of the biomedical literature. J. Am. Coll. Nutr..

[50] Pasin G., Comerford K.B. (2015). Dairy foods and dairy proteins in the management of type 2 diabetes: A systematic review of the clinical evidence. Adv. Nutr..

[51] Appleby P., Roddam A., Allen N., Key T. (2007). Comparative fracture risk in vegetarians and nonvegetarians in EPIC-Oxford. Eur. J. Clin. Nutr..

[52] Vogel K.A., Martin B.R., McCabe L.D., Peacock M., Warden S.J., McCabe G.P., Weaver C.M. (2017). The effect of dairy intake on bone mass and body composition in early pubertal girls and boys: A randomized controlled trial. Am. J. Clin. Nutr..

[53] Merrilees M.J., Smart E.J., Gilchrist N.L., Frampton C., Turner J.G., Hooke E., March R.L., Maguire P. (2000). Effects of diary food supplements on bone mineral density in teenage girls. Eur. J. Nutr..

[54] Avenell A., Mak J.C., O’Connell D. (2014). Vitamin D and vitamin D analogues for preventing fractures in post-menopausal women and older men. Cochrane Database Syst. Rev..

[55] McGrane M.M., Essery E., Obbagy J., Lyon J., MacNeil P., Spahn J., Van Horn L. (2011). Dairy Consumption, Blood Pressure, and Risk of Hypertension: An Evidence-Based Review of Recent Literature. Curr. Cardiovasc. Risk Rep..

[56] Casellas F., Aparici A., Pérez M.J., Rodríguez P. (2016). Perception of lactose intolerance impairs health-related quality of life. Eur. J. Clin. Nutr..

[57] Erminia R., Tiziana M., Antonio N., Loris B., Silvia P., Pierpaolo D., Ilaria B. (2013). HRQoL questionnaire evaluation in lactose intolerant patients with adverse reactions to foods. Intern. Emerg. Med..

[58] Lötters F.J., Lenoir-Wijnkoop I., Fardellone P., Rizzoli R., Rocher E., Poley M.J. (2013). Dairy foods and osteoporosis: An example of assessing the health-economic impact of food products. Osteoporos. Int..

